# Micrandilactone C, a Nortriterpenoid Isolated from Roots of *Schisandra chinensis*, Ameliorates Huntington’s Disease by Inhibiting Microglial STAT3 Pathways

**DOI:** 10.3390/cells12050786

**Published:** 2023-03-02

**Authors:** Minhee Jang, Jong Hee Choi, Dae Sik Jang, Ik-Hyun Cho

**Affiliations:** 1 Department of Convergence Medical Science, College of Korean Medicine, Kyung Hee University, Seoul 02447, Republic of Korea; 2 Department of Pharmaceutical Science, College of Pharmacy, Kyung Hee University, Seoul 02447, Republic of Korea; 3 Institute of Korean Medicine, College of Korean Medicine, Kyung Hee University, Seoul 02447, Republic of Korea

**Keywords:** micrandilactone C, microglia, STAT3, Huntington’s disease, neuroprotection

## Abstract

Huntington’s disease (HD) is a neurodegenerative disease that affects the motor control system of the brain. Its pathological mechanism and therapeutic strategies have not been fully elucidated yet. The neuroprotective value of micrandilactone C (MC), a new schiartane nortriterpenoid isolated from the roots of Schisandra chinensis, is not well-known either. Here, the neuroprotective effects of MC were demonstrated in 3-nitropropionic acid (3-NPA)-treated animal and cell culture models of HD. MC mitigated neurological scores and lethality following 3-NPA treatment, which is associated with decreases in the formation of a lesion area, neuronal death/apoptosis, microglial migration/activation, and mRNA or protein expression of inflammatory mediators in the striatum. MC also inhibited the activation of the signal transducer and activator of transcription 3 (STAT3) in the striatum and microglia after 3-NPA treatment. As expected, decreases in inflammation and STAT3-activation were reproduced in a conditioned medium of lipopolysaccharide-stimulated BV2 cells pretreated with MC. The conditioned medium blocked the reduction in NeuN expression and the enhancement of mutant huntingtin expression in ST*Hdh*^Q111/Q111^ cells. Taken together, MC might alleviate behavioral dysfunction, striatal degeneration, and immune response by inhibiting microglial STAT3 signaling in animal and cell culture models for HD. Thus, MC may be a potential therapeutic strategy for HD.

## 1. Introduction

Huntington’s disease (HD) is a genetic disorder that causes the progressive degeneration of brain cells, particularly in the basal ganglia and cerebral cortex. HD typically causes a combination of chorea, cognitive impairment, and psychiatric symptoms in patients [1,2]. Neurodegeneration in HD is caused by an expansion of a CAG trinucleotide repeat in the huntingtin (*Htt*) gene. The CAG repeat encodes an abnormally long polyglutamine (PolyQ) tract in the huntingtin protein, specifically, striatal medium spiny neurons [1,2]. The abnormal aggregation of mutant huntingtin (mHTT) protein may produce multiple pathological features, including neuronal loss, neuronal toxicity, excitotoxicity, mitochondrial dysfunction, transcriptional dysfunction, changes in axonal transport, and synaptic dysfunction within various brain areas such as the striatum [1,2].

Despite there being many promising theories about the pathological mechanisms underlying HD, there are few pharmacotherapies that have been proven to effectively target these mechanisms and improve symptoms (chorea and psychosis) in clinical trials [3]. Tetrabenazine (Xenazine^®^) is currently the only medication approved by the US Food and Drug Administration for the treatment of HD, and some newer antipsychotic agents (olanzapine and aripiprazole) might have adequate efficacy with a more favorable adverse-effect profile than older antipsychotic agents for treating chorea and psychosis. However, they might produce serious adverse effects such as akathisia, depression, dizziness, and fatigue [3]. Nonetheless, the exact mechanism underlying neuronal death in HD has not been fully elucidated yet.

As a metabolite of 3-nitropropanol, 3-nitropropionic acid (3-NPA) is a naturally occurring toxin that has been found in various fungal species, including *Aspergillus flavus*, *Astragalus*, and *Arthrinium* [4,5]. It can irreversibly inhibit the activity of mitochondrial complex II, also known as succinate dehydrogenase, which is an essential component of both the electron transport chain and the tricarboxylic acid cycle in mitochondria [4,5,6]. Systematically administering 3-NPA into experimental rodent models can cause striatal toxicity. It closely mimics and reproduces behavioral (hyperkinetic and hypokinetic movement), histopathological, and neurochemical pathology features seen in HD [4,5]. Thus, 3-NPA has been used as an efficient chemical to induce HD-like symptoms and pathological features in animal models to study HD [4,5]. The ST*H*dh^Q111/111^ cell line is a striatal cell line derived from a knock-in transgenic mouse containing homozygous huntingtin (HTT) loci with a humanized Exon 1 with 111 polyglutamine repeats. The ST*H*dh^Q111/111^ cell line is a well-known and commonly used model to study molecular aspects of HD [7].

*Schisandra (S.) chinensis,* commonly known as ‘Omija’ in Korean and ‘Wǔ wèi zi’ in Chinese, meaning five-flavor berry, is a plant species that belongs to the genus *Schisandra* of the family Schisandraceae. It is distributed and cultivated in northeastern China, far-eastern Russia, Japan, and Korea [8,9,10]. *S. chinensis* has attracted much attention due to its various pharmacologic effects on different body systems, including the nervous, endocrine, immune, circulatory, and gastrointestinal systems [11]. *S. chinensis* has various compounds, including lignans, nortriterpenes, sesquiterpenes, and phenolic acids [12]. *S.* nortriterpenoids are a structurally intriguing group of polycyclic, highly oxygenated, and fused heterocyclic natural products isolated from *S. chinensis* [12]. We isolated micrandilactone C (MC), a new schiartane nortriterpenoid, from the roots of S. chinensis in our previous study [13]. However, the pharmacological features of MC are not known yet. A previous study has shown that MC isolated from *S. micrantha* exhibits an EC_50_ value of 7.71 µg/mL (SI > 25.94) against human immunodeficiency virus (HIV)-1 replication with minimal cytotoxicity (>200 µg/mL) [14]. A nortriterpenoid kudsuphilactone B isolated from fruits of *S. chinensis* can induce caspase-dependent apoptosis in human cancer cells by regulating Bcl-2 family protein and mitogen-activated protein kinase signaling [15]. C21 nortriterpenoid (16,17-dehydroapplanone E), isolated from *Ganoderma applanatum*, has shown inhibitory effects on the release of nitric oxide (NO) by the lipopolysaccharide (LPS)-induced BV-2 microglial cell line derived from C57/BL6 murine [16]. Novel nortriterpenoid (compound 2) from fruits of *Evodia rutaecarpa* has shown potent neuroprotective activities against serum-deprivation-induced P12 cell damage [17]. These results suggest that MC might have beneficial activities for various pathological statuses including neurological disorders. Herein, we report that MC could ameliorate Huntington’s disease through its anti-inflammatory effects by inhibiting STAT3 pathways.

## 2. Materials and Methods

### 2.1. Animals and Ethical Approval

Male adult C57BL/6 mice (Narabiotec Co., Ltd., Seoul, Republic of Korea; weight: 23–25 g; *n* = 105; seed mice originated from Taconic Biosciences Inc., Rensselaer, NY, USA) were kept under constant temperature (23  ±  2 °C) and humidity (55  ±  5%) conditions with a 12 h light–dark cycle (light on 08:00 to 20:00), and fed food and water ad libitum. The mice were allowed to habituate in the housing facilities for 1 week before the experiments. All experimental procedures were reviewed and approved by the Institutional Animal Care and Use Committee of Kyung Hee University (KHUASP-19-018). In this process, the proper randomization of laboratory animals and handling of data were performed in a blinded manner in accordance with the recent recommendations from a NIH Workshop on preclinical models of neurological diseases [18].

### 2.2. Experimental Group, Model Induction, and Drug Treatment

The experimental group was divided into the following groups: the sham group (vehicle treatment, i.p. +saline, i.v.), 3-NPA group (70 mg/kg of 3-NPA, i.p. +saline, i.v.), 3-NPA + MC 1.25 group (70 mg/kg of 3-NPA, i.p. +1.25 mg/kg of MC, i.v.), 3-NPA + MC 2.5 group (70 mg/kg of 3-NPA, i.p. +2.5 mg/kg of MC, i.v.), and MC alone group (vehicle treatment, i.p. +2.5 mg/kg of MC, i.v.). The 3-NPA model induction was performed according to the method published in [8,19,20,21]. Briefly, 3-NPA (Sigma-Aldrich, St. Louis, MO, USA) was dissolved in saline (25 mg/mL) and passed through a 0.2 µm filter. The 3-NPA was intoxicated intraperitoneally daily for five days at a dose of 70 mg/kg. MC was isolated from roots of *S. chinensis* as previously described [13]. MC was administered daily for five days at one hour before every 3-NPA intoxication.

### 2.3. Behavioral Semi-Quantitative Assessment

The severity of the neurological impairment (motor disability) induced by 3-NPA was assessed by an experimenter who was unaware of the experimental conditions under constant temperature and humidity conditions in a quiet room using the behavioral scale as previously described [8,19,20,21,22,23]. The neurological impairment was evaluated at 24 h after the last (5th) 3-NPA intoxication.

### 2.4. Histopathological Analysis of Striatal Damage

To investigate the histopathological alterations of the striatum following 3-NPA intoxication, we used a previously described protocol [8,22]. Briefly, 24 h after the last (5th) 3-NPA intoxication, the mice (*n* = 5 per group) were anesthetized with isoflurane and then perfused intracardially with saline and iced 4% paraformaldehyde in 0.1 M of phosphate buffer (PB, pH 7.4). Sequential coronal sections (30 μm in thickness) were acquired from the corpus callosum throughout the entire striatum (bregma 1.40~−1.30 mm) using the method published in [24]. Free-floating sections were collected in an antifreeze solution (30% sucrose in PBS) and stored at −20 °C.

### 2.5. Fluoro-Jade C (FJC) and Cresyl Violet Stains

To assess the striatal apoptosis in the striatum after 3-NPA-intoxication, FJC staining was performed using the method published in [25]. Briefly, 24 h after the last (5th) 3-NPA intoxication, free-floating brain sections (3 sections per brain) from all groups (*n* = 5 per group) were immersed in 70% ethyl alcohol, washed with distilled water (DW), and incubated in 0.06% potassium permanganate solution. The sections were washed with DW and then incubated in a solution of 0.001% FJC (Millipore, Billerica, MA, USA). After washing with DW, these sections were air-dried, immersed in 100% xylene, and coverslipped with DPX mountant (Sigma-Aldrich). The region of interest of each section was captured using a confocal laser scanning microscope (LSM 5 PASCAL, Carl Zeiss Microscopy GmbH, Münche, Germany). The number of FJC positive cells per section was manually and blindly counted. Additionally, 3 sections from the level of the mid-striatum were stained with 0.1% cresyl violet dye. Stained sections were captured using a digital camera (DP-70, Olympus Co., Tokyo, Japan). The level of 3-NPA-induced striatal damage compared to the area of the whole striatum was measured using the NIH Image J program [http://rsbweb.nih.gov/ij/ (12 July 2022)].

### 2.6. Immunohistochemical and Immunofluorescence Evaluation

Immunohistochemistry was performed using the method published in [8,22]. Briefly, 24 h after the last (5th) 3-NPA intoxication, free floating brain sections (30 μm thickness; 3 sections per brain) from all groups (*n* = 5 per group) were incubated with rabbit anti-ionized calcium-binding adapter molecule (Iba)-1 (1:2000; WAKO, Chuo-Ku, Japan). The stained sections from the level of the mid-striatum were captured using a digital camera (DP-70, Olympus Co.) and the mean level of Iba-1-immunopositive area to whole striatal area was analyzed using the NIH Image J program [http://rsbweb.nih.gov/ij/ (12 July 2022)] Immunofluorescence analysis was performed as previously described [22,26,27]. Briefly, free floating brain sections (30 μm thickness) from each group (*n* = 5 per group) were blocked with either rabbit anti-phospho (p)-STAT3 (1:200; Cell Signaling Technology, Beverly, MA, USA) and rat anti-CD11b (1:500; Serotec, Oxford, UK). Additionally, the region of interest of each section was captured using a confocal laser scanning microscope (LSM 5 PASCAL, Carl Zeiss, Microscopy GmbH) and the number of p-STAT3^+^ per 500 μm^2^ and the ratio of CD11b (+) cells containing p-STAT3 (+) signal per striatum was manually and blindly measured.

### 2.7. Western Blot Analysis

Western blot analysis was performed using the method published in [8,22]. Briefly, 24 h after the last (5th) 3-NPA intoxication, the striatal proteins from all groups (*n* = 5 per group) were incubated with primary antibodies, including mouse anti-succinate dehydrogenase complex subunit A (SDHA) (1:1000; Abcam, Cambridge, UK), rabbit an-ti-pro-caspase-3 (1:1000; Cell Signaling Technology), rabbit anti-cleaved caspase-3 (1:500; Cell Signaling Technology), rabbit anti-pro-caspase-9 (1:1000; Cell Signaling Technology), rabbit anti-cleaved caspase-9 (1:1000; Cell Signaling Technology), rabbit anti-B-cell lymphoma 2 (Bcl-2) (1:1000; Santa cruz Technology), rabbit anti-Iba-1 (1:500; WAKO), rabbit anti-phospho (p)-STAT3, STAT3 (1:500; Cell Signaling Technology), mouse anti-inducible nitric oxide synthases (iNOS) (1:500; Santa Cruz Biotechnology, Santa Cruz, CA, USA), rabbit anti-interleukin(IL)-1ß, IL-6, tumor necrosis factor-α (TNF-α) (1:1000; Cell Signaling Technology), mouse anti-neuronal nuclear protein (NeuN) (1:2000; Millipore), mouse anti-HTT (*clone* mEM48; 1:500; Millipore), and mouse anti-HTT (clone 2Q75; 1:500; LifeSpan BioSciences, Seattle, WA, USA) antibodies. For the normalization of antibody signals, membranes were stripped and reprobed with antibodies against glyceraldehyde-3-phosphate dehydrogenase (GAPDH; 1:5000; Cell Signaling Technology) or STAT3. Data are expressed as the ratio of the corresponding protein signal against GAPDH or the STAT3s signal for each sample. Original images from Western blot assay in Supplementary Data S1.

### 2.8. Flow Cytometry

At 24 h following the last (5th) 3-NPA intoxication, mice (*n* = 3 per group) with representative behavioral scores in each experimental group were anesthetized by isoflurane (1–2%) and perfused intracardially with saline. The striata were then carefully cropped. To test the microglia/macrophage population, single-cell suspensions refined from striata were prepared and fluorescently stained as previously described [26,28,29,30]. Microglia and macrophages were differentiated based on their relative CD45 expression levels [26,28,29,30]. Briefly, after acquiring unstained and single colored control samples to calculate the compensation matrix, 1 × 10^4^ events were acquired within the combined gate based on physical parameters (forward scatter (FSC) and side scatter (SSC)).

### 2.9. Real-Time Polymerase Chain Reaction (PCR) Analyses

To measure the mRNA level of inflammatory factors, 24 h after the last (5th) 3-NPA intoxication, real-time PCR analysis using the striatal lysats from all groups (*n* = 5 per group) was performed using the SYBR Green PCR Master Mix as previously described [31,32]. Reactions were performed in duplicate in a total volume of 10 μL, each containing 10 pM of primer, 4 μL of cDNA, and 5 μL of SYBR Green PCR Master Mix. The mRNA levels of each target gene were normalized to that of GAPDH mRNA. Fold-induction was calculated using the 2^−ΔΔCT^ method as previously described [33]. All real-time RT–PCR experiments were performed at least three times, and the mean ± SEM values are presented unless otherwise noted. The primer sequences are listed in Supplementary Materials. The expression levels of each gene were normalized to that of GAPDH.

### 2.10. STHdh Cell Culture

ST*Hdh* cell lines (ST*Hdh*^Q111/Q111^) (conditionally immortalized striatal neuron progenitor cell lines) were kindly provided by Prof. Hoon Ryu (Korea Institute of Science and Technology, Seoul, Republic of Korea) and were cultured according to the protocol from Coriell Institute for Medical Research (Camden, NJ, USA) as previously described [22].

### 2.11. Preparation of Conditioned Medium (CM) from BV2 Cells and Determination of Activity of STHdh Cells

To obtain CM, cultured BV2 cells were treated with MC (5 μM) at 1 h before stimulation with 3-NPA (1 mM) for 12 h. The culture medium was replaced with fresh medium and incubated for 24 h. CM-3-NPA (conditioned medium from 3-NPA-stimulated BV2 cells) and CM-3-NPA-MC (conditioned medium from 3-NPA-stimulated BV2 cells pretreated with MC) were collected and used to investigate the expression of inflammatory factors and p-STAT3 by Western blot analysis. CM-3-NPA and CM-3-NPA-MC were treated to ST*Hdh*^Q111/Q111^ cells for 24 h. CM-treated ST*Hdh*^Q111/Q111^ cells were collected to analyze the degree of neurodegeneration (NeuN) and huntingtin aggregation (EM48 and 2Q75) by Western blot analysis. In vitro assays were repeated at least three times, with each experiment performed in triplicate.

### 2.12. Statistical Analysis

Statistical analysis was performed using the IBM SPSS Statistics Version 26.0 (SPSS Inc., Chicago, IL, USA) for Windows. The data from experiments including the behavioral test, immunohistochemistry, Western blot, and PCR analysis were analyzed using Kruskal–Wallis test (a nonparametric test) for the comparison of three or more unmatched groups. The data are presented as mean ± SEM. *p* values of less than 0.05 were accepted as statistically significant.

## 3. Results

### 3.1. Effects of MC on Neurological Score and Survival Rate after 3-NPA Intoxication

**Figure 1:**
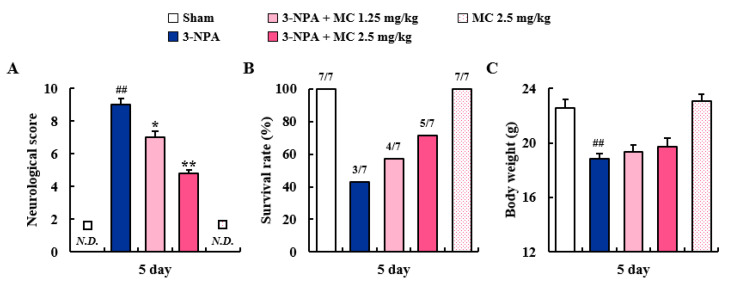
MC alleviates neurological dysfunction and improves survival rate after 3-NPA treatment. (**A**–**C**) Effects of MC on neurological score (motor disability), survival rate, and BW during 3-NPA treatment period. MC was intravenously treated once daily for 5 days 1 h before 3-NPA treatment. At 24 h after the last (5th) 3-NPA treatment, the neurological score (**A**), survival rate (**B**), and BW (**C**) of mice from sham (*n* = 7), 3-NPA (*n* = 7), 3-NPA + MC (1.25 mg/kg/day; *n* = 7), 3-NPA + MC (2.5 mg/kg/day; *n* = 7) and MC (2.5 mg/kg/day; *n* = 7) groups were measured. For the neurological score, levels of global activity, hindlimb clasping, hindlimb dystonia, truncal dystonia, and balance adjustment to a postural challenge were measured and their values were combined. N.D., not detected. In graph B, the values above the bars represent survival rates (number of surviving animals/number of total animals). Data are expressed as mean ± standard error of the mean (SEM) (Kruskal–Wallis; ## *p* < 0.01 versus sham group; * *p* < 0.05 and ** *p* < 0.01 versus 3-NPA group).

First, we determined whether MC could mitigate neurological signs and survival rate of mice following 3-NPA treatment. Figure 1A–C shows a representative neurological score, survival rate, and body weight (BW) of the sham, 3-NPA, 3-NPA + MC (1.25 and 2.5 mg/kg/day), and MC alone (2.5 mg/kg/day) groups. Twenty-four hours after the last (5th) intoxication of 3-NPA, the mice displayed symptoms of severe neurological deficits (score, 9.0 ± 0.4). However, the mice in 3-NPA + MC groups displayed significantly lower neurological scores (7.0 ± 0.4 and 4.8 ± 0.2 in MC 1.25 and 2.5 mg/kg/day groups, respectively) than the mice in the 3-NPA group (score, 9.0 ± 0.4) (Figure 1A). The survival rate at the end of the representative experimental set was increased to 57.1% (*n* = 4/7) and 71.4% (*n* = 5/7), respectively, in 3-NPA + MC 1.25 mg/kg/day and 3-NPA + MC 2.5 mg/kg/day groups, respectively, as compared to that in the 3-NPA group (42.8%, *n* = 3/7) (Figure 1B). The mean loss of BW was significantly alleviated by 3-NPA. However, it was not significantly affected by MC treatment at 1.25 or 2.5 mg/kg/day (Figure 1C). Treatment with MC alone (2.5 mg/kg/day) did not significantly affect the neurological score, survival rate, or BW of normal mice.

### 3.2. Effects of MC on Striatal Cell Death and Apoptosis Induced Following 3-NPA-Treatment

**Figure 2:**
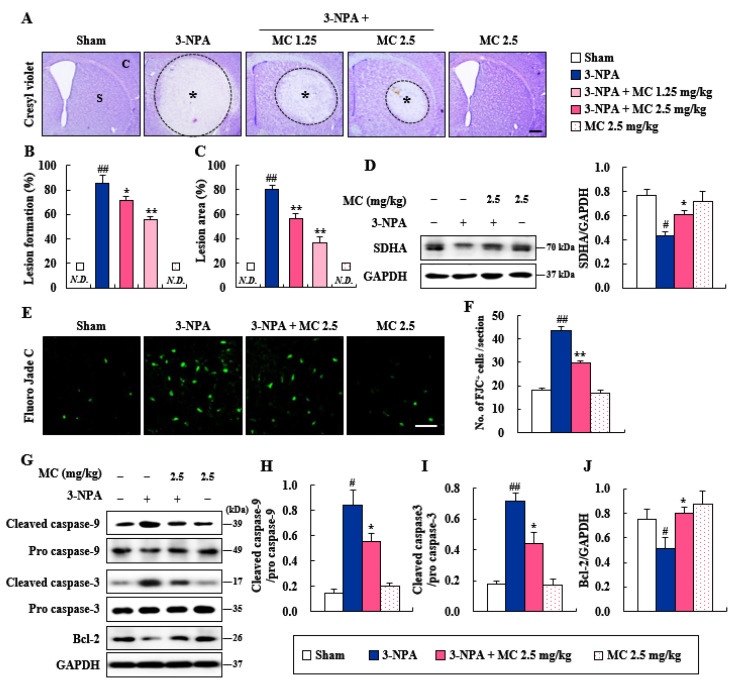
MC prevents neurodegeneration and apoptosis in the striatum after 3-NPA treatment. (**A**–**F**) At 24 h following the last (5th) 3-NPA treatment, striata from sham (*n* = 5), 3-NPA (*n* = 5), 3-NPA + MC (2.5 mg/kg/day; *n* = 5), and MC (2.5 mg/kg/day; *n* = 5) groups were subjected to histopathological and Western blot analyses to investigate the effect of MC (2.5 mg/kg/day, i.v.) on striatal cell death and apoptosis. (**A**–**C**) Representative photographs showing the level of striatal lesion by cresyl violet staining (**A**). MC significantly reduced the number of mice with striatal lesion (**B**) and the lesion area (**C**). Asterisk, S, and C indicate striatal lesions, normal striatum, and normal cortex, respectively. Scale bar = 100 μm. N.D., not detected. Data are expressed as mean ± SEM (Kruskal–Wallis; # *p* < 0.05 and ## *p* < 0.01 versus sham group; * *p* < 0.05 and ** *p* < 0.01 versus 3-NPA group). (**D**,**F**,**G**) Striata from all groups were analyzed by Western blot to determine the expression level of SDHA (**D**), cleaved caspase-9 (**G**,**H**), cleaved caspase-3 (**G**,**I**), and Bcl-2 (**G**,**J**). MC prevented the enhancement of these protein expressions. (**E**,**F**) Representative photographs (**E**) and quantified graph (**F**) showing the level of striatal cell death by FJC staining. MC significantly reduced the number of FJC (+) cells. Scale bar = 200 μm. Data are expressed as mean ± SEM (Kruskal–Wallis; ## *p* < 0.01 versus sham group; * *p* < 0.05 and ** *p* < 0.01 versus 3-NPA group).

It is known that 3-NPA-induced neurological dysfunction results from striatal cell death [19,20,21,23]. Thus, we explored whether MC could alleviate striatal cell death following 3-NPA-treatment. Twenty-four hours after the last (5th) 3-NPA treatment, coronal *cryostat sections* of brain including the striatum were subjected to cresyl violet dye (Figure 2A). Figure 2A shows representative striatal images from the sham, 3-NPA, 3-NPA + MC (1.25 and 2.5 mg/kg/day), and MC alone (2.5 mg/kg/day) groups. In the two representative experimental sets, it was found that 85.7% (*n* = 6/7) of the surviving mice in the 3-NPA-treated group had visible bilateral striatal lesions (pale areas surrounded by dotted line), whereas this percentage was reduced to 71.4% (*n* = 5/7) and 55.5% (*n* = 5/9) in the groups treated with MC at 1.25 and 2.5 mg/kg/day, respectively (Figure 2B). Furthermore, in the 3-NPA group, the ratio of the mean lesion area to the entire striatum was 80.6%, whereas this ratio remarkably decreased to 56.1% and 36.6% in the group treated with MC at 1.25 and 2.5 mg/kg/day, respectively (Figure 2C). The results of the behavioral dysfunction (Figure 1A) and striatal cell death (Figure 2A–C) revealed that treatment with 2.5 mg/kg/day of MC was more effective in inhibiting 3-NPA toxicity than treatment with 1.25 mg/kg/day of MC. Thus, the dose of 2.5 mg/kg/day of MC was used in further studies. Since 3-NPA is an irreversible inhibitor of mitochondrial respiratory complex II and succinate dehydrogenase (SDH) [4,5,6], we explored whether MC could inhibit mitochondrial complex II activity using SDHA antibody in striatal lysate at 24 h after the last 3-NPA administration (Figure 2D). Protein expression level of SDHA was decreased in the 3-NPA group (0.43) compared to that in the sham group (0.77) but increased after treatment with MC at 2.5 mg/kg/day (0.61) (Figure 2D). Based on the results from the cresyl violet stain (Figure 2A–C), to further compare the levels of degenerating neuronal cells, we stained coronal *cryostat sections* with FJC anionic fluorescent dye (Figure 2E,F), a good marker of degenerating neurons [34,35]. The number of FJC (+) cells was increased to 43.6 ± 1.5 per section in the 3-NPA group, but decreased to 29.6 ± 0.9 in the 3-NPA + 2.5 mg/kg/day MC group (Figure 2E,F). To test whether the anti-neuronal cell death effect of MC might be related to apoptosis, we determined the protein levels of the representative apoptosis markers (cleaved caspase-9, cleaved caspase-3, and Bcl-2) in the striatum by Western blotting (Figure 2G–J). The protein expression levels of cleaved caspase-9 and cleaved caspase-3 were increased in the 3-NPA group (0.85 and 0.71, respectively) compared to those in the sham group (0.15 and 0.17, respectively), but decreased after treatment with MC at 2.5 mg/kg/day (0.55 and 0.44, respectively) (Figure 2G–J), similar to results of the FJC staining (Figure 2E,F). The protein expression level of Bcl-2 was also decreased in the 3-NPA group (0.51) compared to that in the sham group (0.75) but increased after treatment with MC at 2.5 mg/kg/day (0.80) (Figure 2G–J).

### 3.3. Effect of MC on Microglial Activation in the Striatum Following 3-NPA-Treatment

**Figure 3:**
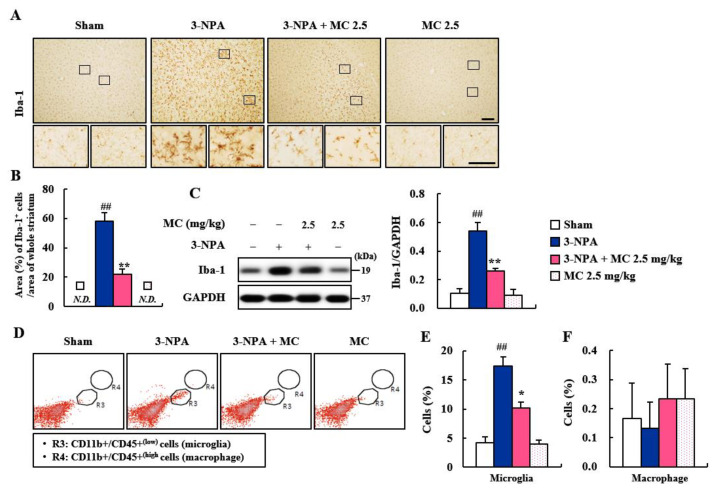
MC inhibits microglial migration and activation in striatum after 3-NPA treatment. (**A**–**F**) Twenty-four hours after the last (5th) 3-NPA treatment, striata from sham, 3-NPA, 3-NPA + MC (2.5 mg/kg/day), and MC (2.5 mg/kg/day) groups were used to investigate the levels of migration of microglia and infiltration of macrophages. MC prevented the migration and activation of Iba-1 immunoreactive cells by immunohistochemistry ((**A**); *n* = 5 per group), the mean area of Iba-1 immunoreactive cells ((**B**): *n* = 5 per group)’ the expression of Iba-1 protein by Western blot assay ((**C**): *n* = 5 group). MC reduced the level of migration of resident microglia (R3; CD11b^+^/CD45^+low^; (**D**,**E**)) by flow cytometry (*n* = 3 per group), but did not significantly affect the infiltration of peripheral macrophage (R4; CD11b^+^/CD45^+high^; (**D**,**F**)). Scale bar = 100 μm. The small box inside the upper panel of a was enlarged on the lower panel. N.D., not detected. Data are expressed as mean ± SEM (Kruskal–Wallis; ## *p* < 0.01 versus sham group; * *p* < 0.05 and ** *p* < 0.01 versus 3-NPA group).

Microglia are migrated into degenerative site in the central nervous system (CNS) in cases of neurodegenerative diseases, including HD. They are then activated within/around the lesions in the CNS. These activated microglia can produce pro- and anti-inflammatory cytokines [36,37,38]. Thus, we explored whether MC could suppress microglial activation in the striatal lesions from all groups (*n* = 5 per group) following 3-NPA treatment (Figure 3A–C and Supplementary Data S2). In the striatal sections of the 3-NPA group, Iba-1 (a marker for microglia/macrophage lineage cells)-immunoreactive cells showed a morphology of the activated type with bigger cell bodies and extended (short and thick) processes than those in the sham group of CNS, which displayed typical forms of resting cells, including relatively small soma and long, thin processes [19,20,21,36] (Figure 3A–C). However, the mean level of Iba-1-immunopositive area to whole striatal area was clearly decreased in striatal sections of the 3-NPA + MC group than in the 3-NPA group (Figure 3A,B), in agreement with the alteration (0.53-fold in the 3-NPA group; 0.26-fold in the MC) in the protein expression of Iba-1 based on Western blot analysis (Figure 3C). The morphology of the Iba-1 immunoreactive cells based on immunohistochemistry and the Iba-1 protein expression based on Western blot analysis were not significantly affected by treatment with MC (2.5 mg/kg/day) alone (Figure 3A–C). Since Iba-1 can detect microglia and macrophage [19,20,21,36]; to discriminate both cells, flow cytometry was performed using striatum at 24 h after the last (5th) treatment of 3-NPA. Interestingly, the percentage of CD11b^+^/CD45^+(low)^ cells representing microglial cells increased to 17.4 ± 1.1% in the 3-NPA group compared to that of the sham group (4.2 ± 0.6%) but decreased to 10.2 ± 0.5% in the 3-NPA + MC group compared to that of the 3-NPA group (Figure 3D,E). However, the percentage of CD11b^+^/CD45^+(high)^ cells representing macrophages was not significantly different between the sham group and the other groups (Figure 3D,F). These findings suggest that MC might inhibit microglial migration and activation regardless of the macrophage and that MC might be closely associated with the reduction in striatal cell death and the mitigation of neurological impairment following 3-NPA treatment.

### 3.4. Effects of MC on Inflammatory Factors and STAT3 Pathways in the Striatum Following 3-NPA-Treatment

**Figure 4:**
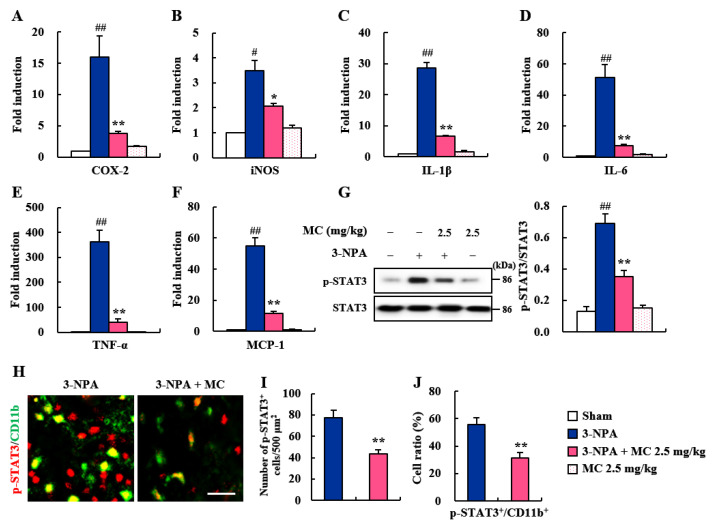
MC reduces the mRNA or protein expression levels of inflammatory factors and p-STAT3 in striatum or microglia after 3-NPA treatment. (**A**–**F**) Twelve hours after the last (5th) 3-NPA-treatment, striatal tissues from sham (*n* = 5), 3-NPA (*n* = 5), 3-NPA + MC (2.5 mg/kg/day; *n* = 5), and MC (2.5 mg/kg/day; *n* = 5) groups were analyzed by real-time PCR. MC reduced mRNA expression levels of representative inflammatory enzymes (COX-2 (**A**) and iNOS (**B**)), cytokines (IL-1β (**C**), IL-6 (**D**), and TNF-α (**E**)), and chemokine (MCP-1 (**F**)). (**G**) Twenty-four hours after the last (5th) 3-NPA-treatmentn, striatal laysats from all groups (*n* = 5 group) were subjected to Western blot analysis. MC reduced protein expression of p-STAT3. Data are expressed as mean ± SEM (Kruskal–Wallis; # *p* < 0.05 and ## *p* < 0.01 versus sham group; * *p* < 0.05 and ** *p* < 0.01 versus 3-NPA group). (**H**–**J**) Twenty-four hours after the last (5th) 3-NPA-treatment, 3 striatal sections from all groups (*n* = 5) were subjected to immunofluorescent staining using mixtures of p-STAT3/CD11b antibodies. Red, green, and yellow colors show p-STAT3^+^, CD11b^+^, and p-STAT3^+^/CD11b^+^, respectively (**H**). The numbers of p-STAT3 (+) cells per 500 μm^2^ and the ratio of CD11b (+) cells containing p-STAT3 (+) signal were measured (**I**,**J**). MC reduced p-STAT3-immunoreactivity in the striatum and CD11b (+) cells. Scale bar = 200 μm. Data are expressed as mean ± SEM (Kruskal–Wallis; ** *p* < 0.01 versus 3-NPA group).

Migrated and activated microglia around (or within) CNS lesions can release inflammatory mediators (enzymes, cytokines, and chemokines) that are either beneficial or detrimental to neuronal survival [36,37,38]. Thus, we explored whether the inhibition of microglial activation by MC might induce changes in the mRNA expression of representative inflammatory enzymes (COX-2 and iNOS), cytokines (IL-1β, IL-6, and TNF-α), and chemokine (MCP-1) using real-time PCR analysis (Figure 4A–F). The mRNA expression levels of pro-inflammatory factors were increased in the 3-NPA group compared to the sham group, with the following results: COX-2: increase by 15.9-fold; iNOS: increased by 3.5-fold; IL-1β: increased by 28.7-fold; IL-6: increased by 51.4-fold; TNF-α: increased by 55.1-fold; and MCP-1: increased by 363.8-fold (Figure 4A–F). On the other hand, MC remarkably blocked these increases induced by 3-NPA with the following results: COX-2 by 8.0%, iNOS, by 3.5%, IL-1β, by 28.7%, IL-6, by 51.4%, TNF-α, by 55.1%, and MCP-1 by 89.0%, compared to those in the 3-NPA group (Figure 4A–F). Since STAT3 pathways are involved in neurodegeneration, including striatal toxicity [39,40], we examined these signaling pathways in the striatum after 3-NPA treatment (Figure 4G–J). The expression level of p-STAT3 protein was remarkably enhanced—by 5.3-fold—in the striatum at 24 h after the final 3-NPA treatment compared to that in the sham group. However, MC significantly inhibited the expression level of p-STAT3 protein by 49.3% (Figure 4G). To determine whether STAT3 downregulation by MC was directly related to the reduction in neuronal cell death and microglial activation, we performed immunofluorescence staining for p-STAT3 in the striatum of the 3-NPA group. In agreement with the alteration in the expression level of p-STAT3 protein, the numbers of p-STAT3 immunoreactive cells and CD11b (+) cells were enhanced in striatal lesions after 3-NPA treatment, while these numbers were markedly reduced by MC treatment (Figure 4H–J). These findings suggest that MC could inhibit inflammatory response and striatal toxicity after 3-NPA treatment by inhibiting STAT3 pathways in the striatum and microglia.

### 3.5. Effects of MC on Pro-Inflammatory Factors and STAT3 Pathways in 3-NPA-Induced BV2 Cells

**Figure 5:**
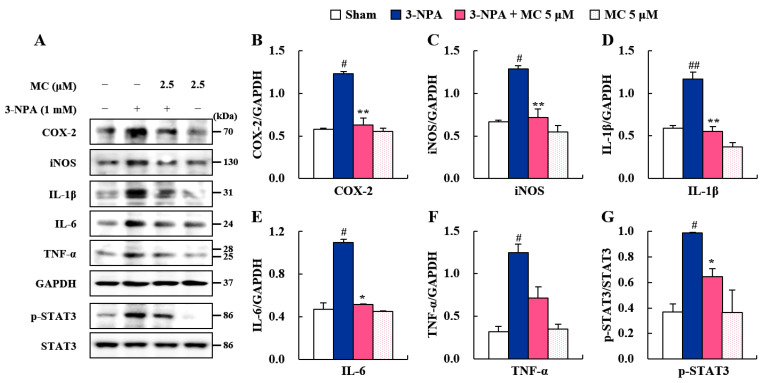
MC reduces the expression of inflammatory factors and p-STAT3 in 3-NPA-induced BV2 cells. (**A**–**G**) Cultured BV2 cells were treated with MC (5 μM) at 1 h before stimulation with 3-nitropropionic acid (3-NPA) (1.0 mM). Culture medium was replaced with fresh medium and incubated for 12 h. BV2 cells from sham, 3-NPA, 3-NPA + MC (5 μM), and MC (5 μM) groups were used for Western blot analysis to determine protein expression levels of representative inflammatory enzymes (COX-2 (**A**,**B**) and iNOS (**A**,**C**)), cytokines (IL-1β (**A**,**D**), IL-6 (A and E), and TNF-α (**A**,**F**)), and p-STAT3 (**A**,**G**). MC alleviated their expression levels (**A**–**G**). Assays were repeated at least three times, with each experiment performed in triplicate. Data are expressed as mean ± standard error of the mean (Kruskal–Wallis; # *p* < 0.05 and ## *p* < 0.01 versus sham group; * *p* < 0.05 and ** *p* < 0.01 versus 3-NPA group).

The STAT3 pathway plays an important role in microglial activation [41]. Microglial activation is pivotally involved in neuroinflammatory and neurodegenerative events processes such as 3-NPA-induced striatal toxic, adeno-associated viruses (AAV)/viral vector-induced, and transgenic mice models for HD [19,39,42]. Thus, we further investigated whether MC could control microglial activation in 3-NPA-induced BV-2 cell (Figure 5). MC significantly inhibited the enhancement in protein expression of a representative inflammatory enzyme (COX-2 and iNOS) and cytokines (IL-1β, IL-6, and TNF-α) as found using Western blot analysis: COX-2 by 48.7%, iNOS by 44.1%, IL-1β by 52.1%, IL-6 by 53.6%, and TNF-α by 43.2%, compared to those in the 3-NPA-treated group (Figure 5A–F). Next, we investigated whether these anti-inflammatory effects of MC were related to the reduced expression of p-STAT3. The expression of p-STAT3 was markedly enhanced in 3-NPA-stimulated BV2 cells (by 267.5%), compared to those in the sham group. However, MC impressively inhibited this enhancement (by 35.4%) (Figure 5A,G). MC itself did not significantly affect inflammatory enzyme/cytokines and STAT3 phosphorylation (Figure 5A,G). These results suggest that MC might inhibit STAT3 pathways and contribute to microglial downregulation as well as neuroprotection.

### 3.6. Effect of MC on STHdh Cell Death via Microglial Downregulation by Inhibiting STAT3 Pathway

**Figure 6:**
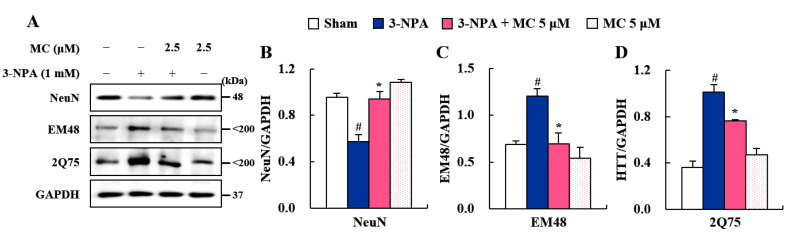
MC enhances the activity of ST*Hdh* cells by inhibiting the expression of p-STAT3 in BV2 cells. (**A**–**D**) To obtain conditioned medium (CM) treated with MC, cultured BV2 cells were treated with MC (5 μM) before stimulation with 3-nitropropionic acid (3-NPA) (1.0 mM, 12 h). CM-3-NPA and CM-3-NPA-MC were used to treat ST*H*dhQ111/Q111 (mutant) cells. ST*H*dh cells were used for Western blot analysis to evaluate neurodegeneration using NeuN antibody (**A**,**B**) and huntingtin aggregation using EM48 and 2Q75 antibodies (**A**,**C**,**D**). Data are expressed as mean ± SEM (Kruskal–Wallis; # *p* < 0.05 vs. vehicle-treated Sham group; * *p* < 0.05 vs. 3-NPA-induced group).

Since the STAT3 pathway plays a critical role in neuron–microglia interactions [41], we further investigated whether these anti-inflammatory effects of MC could affect striatal cell death via the STAT3 pathway by controlling mHTT expression in HD (Figure 6). Impressively, CM-3-NPA significantly reduced the expression of NeuN protein (a marker of neuronal cells) in ST*Hdh*^Q111/Q111^ cells compared to the sham control. However, CM-3-NPA-MC significantly inhibited this reduction (Figure 6A,B). CM-3-NPA also enhanced the expression of EM48 and 2Q75 proteins (markers of mHTT) in the ST*Hdh*^Q111/Q111^ cell compared to the sham control, whereas CM-3-NPA-MC intriguingly diminished their expression levels (Figure 6A,C,D). These results indicate that MC might decrease the ST*Hdh*^Q111/Q111^ cell death related to the reduced expression of mHTT protein by down-regulating microglial activation.

## 4. Discussion

The results of the present study revealed that MC, a nortriterpenoid isolated from roots of *S. chinensis*, could ameliorate 3-NPA-induced HD-like symptoms by inhibiting STAT3 pathways. Pretreatment with MC ameliorated the neurobehavioral disorder (motor disability), improved the survival rate, and inhibited the neurodegeneration related to apoptosis in the striatum following 3-NPA intoxication. These results were consistent with the reduction in microglial activation and inflammatory response related to the reduction in p-STAT3 expression. Intriguingly, CM-3-NPA-MC reduced ST*Hdh*^Q111/Q111^ cell death by inhibiting mHTT expression. These beneficial activities of MC for HD-like symptoms were associated with the inhibition of microglial STAT3 pathways. In conclusion, MC might be a potential therapeutic agent for treating HD-like symptoms by inhibiting microglial STAT3 pathways. To the best of our knowledge, this effect of MC on neurological disorders has never been reported.

An inhibitor of SDH (mitochondrial complex II), 3-NPA is a source of reactive oxygen species [4,5,6]. It is known that 3-NPA can induce striatal degeneration by neurotoxic activity in rodents and result in gait abnormalities, which mimics the behavioral dysfunction and pathology caused by mutant *Htt* in animal models for HD and its patients. However, the 3-NPA-induced rodent model has nothing to do with mutant *Htt* expression [5,43]. Nevertheless, the model has been used to discover a therapeutic intervention for HD [5,43]. In the present study, the protein expression level of SDHA, a marker of mitochondrial complex II activity, was decreased in the striatum following 3-NPA treatment but enhanced by administration with MC (Figure 2). The enhancement of the mitochondrial complex II activity of MC was associated with decreased levels of behavioral impairment (Figure 1) and striatal cell death based on cresyl violet and FJC staining (Figure 2). Taken together, these results suggest that regulating mitochondrial complex II activity might be an attractive strategy to prevent striatal degeneration in 3-NPA-induced HD-like symptoms.

FJC staining is commonly used to label all degenerating mature neurons, including apoptotic, necrotic, and autophagic cells in brain tissue [34,35]. MC blocked the increase in the number of FJC (+) cells in the striatum induced by 3-NPA (Figure 2) associated with reduced levels of cleaved caspase-9/caspase-3 proteins (initiators of intrinsic apoptosis) and enhanced levels of Bcl-2 protein (regulator proteins of apoptosis) (Figure 2). These results suggest an anti-apoptotic activity of MC in striatal degeneration. Normally, 3-NPA can induce apoptosis by generating superoxide radicals [44] and activating the microglia surrounding apoptotic cells [45]. Cell death caused by the latter is called ‘secondary cell death’ or ‘delayed cell death’ [46]. STAT3-activation in microglia exacerbates neuronal apoptosis in the hippocampus of diabetic brains [47]. Thus, controlling microglial STAT3 is considered an attractive anti-apoptosis strategy to protect neurons in various pathological environments. In the present study, MC inhibited the expression of pro-inflammatory factors and STAT3 pathways in 3-NPA-induced BV2 cells (Figure 5). CM-3-NPA-MC significantly reduced ST*Hdh*^Q111/Q111^ cell death (NeuN) associated with mHTT expression (EM48 and 2Q75) (Figure 6). Taken together, these results suggest an anti-apoptotic activity of MC in striatal degeneration by inhibiting microglial STAT3 signaling.

Microglia, as brain-resident immune cells, are emerging as a central player in regulating the key pathways in CNS inflammation [48,49]. Microglia are recruited and activated around or within neurodegenerative lesions. Activated microglia can secrete inflammatory agents that are either beneficial or deleterious to neuronal survival [37]. Clinical studies using positron emission tomography have also demonstrated that the level of microglial activation is increased in proportion to the severity of HD symptoms [38,42]. Thus, handling microglial activation might be an attractive therapeutic strategy for neurological disorders including HD [37]. In the present study, MC inhibited microglial activation (Iba-1 immunoreactive cells) and decreased the mRNA or protein expression levels of pro-inflammatory enzymes (COX-2 and iNOS), cytokines (IL-1β, IL-6, and TNF-α), and chemokine (MCP-1) in the striata of 3-NPA-intoxicated mice and in 3-NPA-induced BV2 cells (Figure 3, Figure 4 and Figure 5). Thus, MC might inhibit microglial activation and inflammatory responses, leading to a reduction in striatal cell death.

STAT3 is a pivotal transcription factor for microglial activation and cytokine production [41,50], such as IL-1β [51], IL-6 [52], and TNF-*α* [53,54]. These cytokines have been identified as important mediators of microglia–neuron interaction during neurodegeneration [55]. The STAT3 signaling pathway is critically involved in behavioral dysfunction and the pathological events of HD and AD [40]. Thus, in this study, we hypothesized that STAT3 signaling in microglia might affect microglia–neuron interactions via secreted cytokines, resulting in striatal degeneration and behavioral dysfunction. As a result of testing this hypothesis, MC inhibited the mRNA or protein expression of representative inflammatory enzyme (COX-2 and iNOS), cytokines (IL-1β, IL-6, and TNF-*α*), and p-STAT3 in not only 3-NPA-intoxicated striatum, but also 3-NPA-stimulated BV2 cells (Figure 3, Figure 4 and Figure 5). MC also inhibited the level of co-staining of p-STAT3 in CD11b positive cells in striatum after 3-NPA-intoxication and protein expression of p-STAT3 in 3-NPA-stimulated BV2 cells (Figure 4 and Figure 5). These findings indicate that MC might reduce inflammatory responses by inhibiting STAT3 signaling in microglia. Furthermore, we investigated whether the downregulation of microglial p-STAT3 might affect the survival of ST*Hdh*^Q111/Q111^ cells expressing mHTT. Interestingly, CM-3-NPA-MC (conditioned medium from 3-NPA-stimulated BV2 cells pretreated with MC) significantly reduced ST*Hdh*^Q111/Q111^ cell death and mHTT expression compared to CM-3-NPA treatment (Figure 6). Taken together, MC might reduce striatal degeneration and mHTT expression through reduced inflammatory responses by inhibiting microglial STAT3 signaling.

Although the mechanisms involve in the anti-inflammatory effects of MC have not yet been reported, such effects might be indirectly explained by the positive effects of representative norteripenoids. For example, C21 nortriterpenoid (16,17-dehydroapplanone E), isolated from *Ganoderma applanatum*, can inhibit the secretion of NO in 3-NPA-induced BV-2 cells [16]. Ulmoidol, an unusual nortriterpenoid from *Eucommia ulmoides* Oliv. leaves, can suppress the production of proinflammatory mediators (TNF-α, IL-1β, IL-1, and PGE2) and reduce the expression of iNOS and COX-2 in 3-NPA-treated BV-2 cells [56]. Additionally, a nortriterpenoid (compound 2) from the fruits of *Evodia rutaecarpa* shows neuroprotective activities against serum-deprivation-induced P12 cell damage [17]. Taken together, these findings suggest that MC from *S. chinensis* might possess remarkable anti-inflammatory activity, which improves the neurological disorders associated with HD-like symptoms.

## 5. Conclusions

The exact mechanism underlying neuronal death and valuable therapeutics in HD-like symptoms has not yet been fully elucidated. Here, we found that MC could mitigate the striatal degeneration related to reduced inflammatory response and mHTT expression by inhibiting STAT3 signaling in microglia. Despite the relative lack of information on the efficacy and critical mechanisms of action of MC, our findings indicate that MC might be used as a potential therapeutic to improve HD-like symptoms by regulating the microglial STAT3 pathways. We also propose that it is necessary to determine the efficacy and mechanisms of action of MC in various pathological conditions, including neurological disease, in future works, in addition to identifying its chemical interactions in vivo.

## Data Availability

The data for this study are available from the corresponding authors upon reasonable request.

## Supplementary Materials

The following supporting information can be downloaded at: https://www.mdpi.com/article/10.3390/cells12050786/s1, Supplementary Materials: Primer sequences used for real-time polymerase chain reaction (PCR) analyses; Supplementary Data S1: Original images from Western blot assay. Supplementary Data S2: MC inhibits microglial migration and activation in striatum after 3-NPA treatment (a low magnification)
