# Supplementary material for: Micrandilactone C, a Nortriterpenoid Isolated from Roots of Schisandra chinensis, Ameliorates Huntington’s Disease by Inhibiting Microglial STAT3 Pathways
Source: Cells. 2023 Mar 2;12(5):786. doi: 10.3390/cells12050786 (PMC10000367; doi:10.3390/cells12050786)
Supplement: Supplementary file 1 [file cells-12-00786-s001.zip › cells-1942380-supplementary.pdf]

Supplementary Materials 1

Primer sequences used for real-time polymerase chain reaction (PCR) analyses

Primer sequences used for PCR analyses were as follows; IL-1β-5′-TTG TGG CTG TGG AGA AGC TGT-3′ and 5′-AAC GTC ACA CAC CAG CAG GTT-3′, IL-6-5′-TCC ATC CAG TTG CCT TCT TGG-3′ and 5′-CCA CGA TTT CCC AGA GAA CAT G-3′, tumor necrosis factor (TNF)-α-5′-AGC AAA CCA CCA AGT GGA GGA-3′ and 5′-GCT GGC ACC ACT AGT TGG TTG T-3′, Cyclooxygenase (COX)-2-5′-CAG TAT CAG AAC CGC ATT GCC-3′ and 5′-GAG CAA GTC CGT GTT CAA GGA-3′, iNOS- 5′-GGC AAA CCC AAG GTC TAG GTT-3′ and 5′-TCG CTC AAG TTC AGC TTG GT-3′, MCP-1-5′-CTT CTG GGC CTG CTG TTC A-3′ and 5′-CCA GCC TAC TCA TTG GGA TCA-3′, IL-4-5′-GAA TGT ACC AGG AGC CAT ATC-3′ and 5′-CTC AGT ACT ACG AGT AAT CCA-3′, IL-10-5′-ATA ACT GCA CCC ACT TCC CA-3′ and 5′-TCA TTT CCG ATA AGG CTT GG-3′, and GAPDH-5′-AGG TCA TCC CAG AGC TGA ACG-3′ and 5′-CAC CCT GTT GCT GTA GCC GTA T-3′.

Supplementary Data S1

Figure 5A

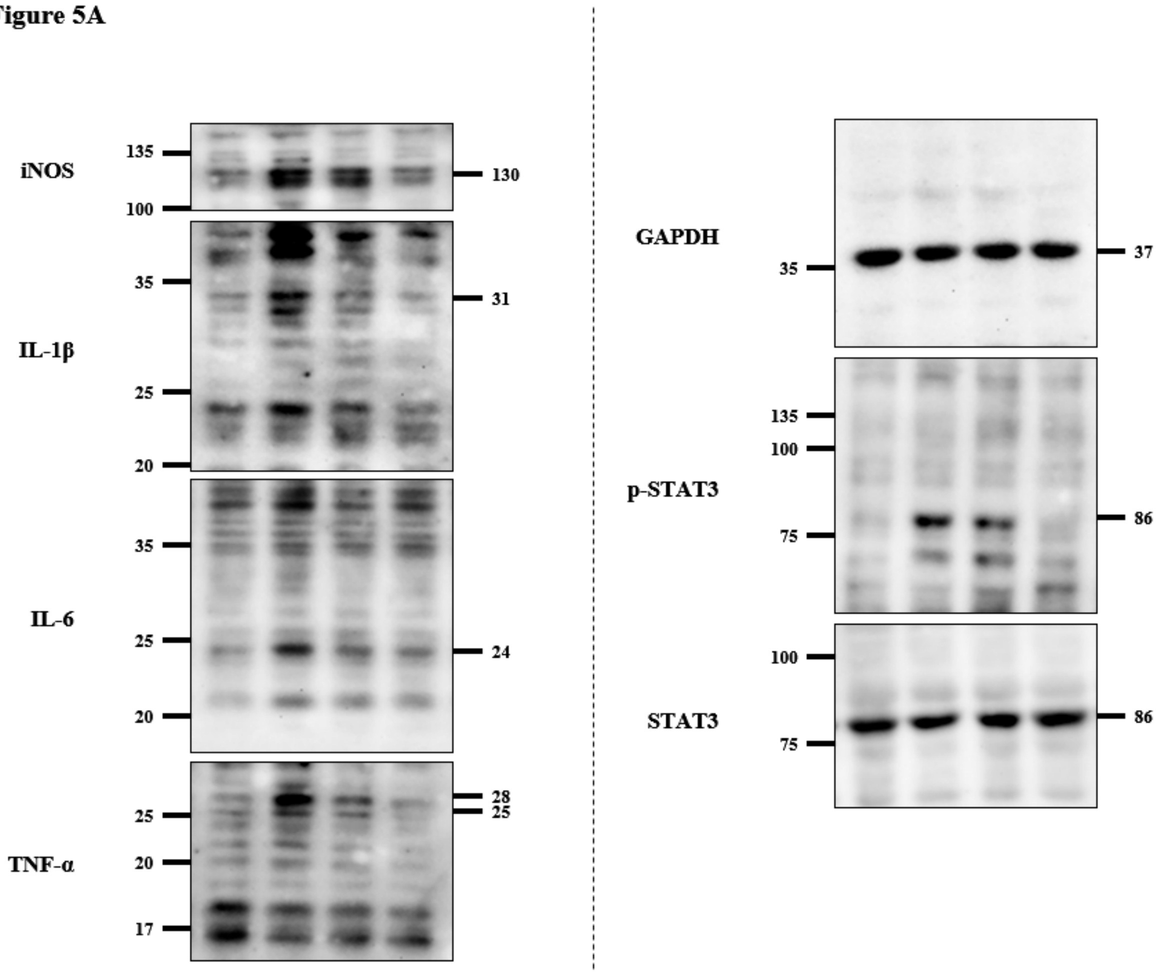

Figure 5G

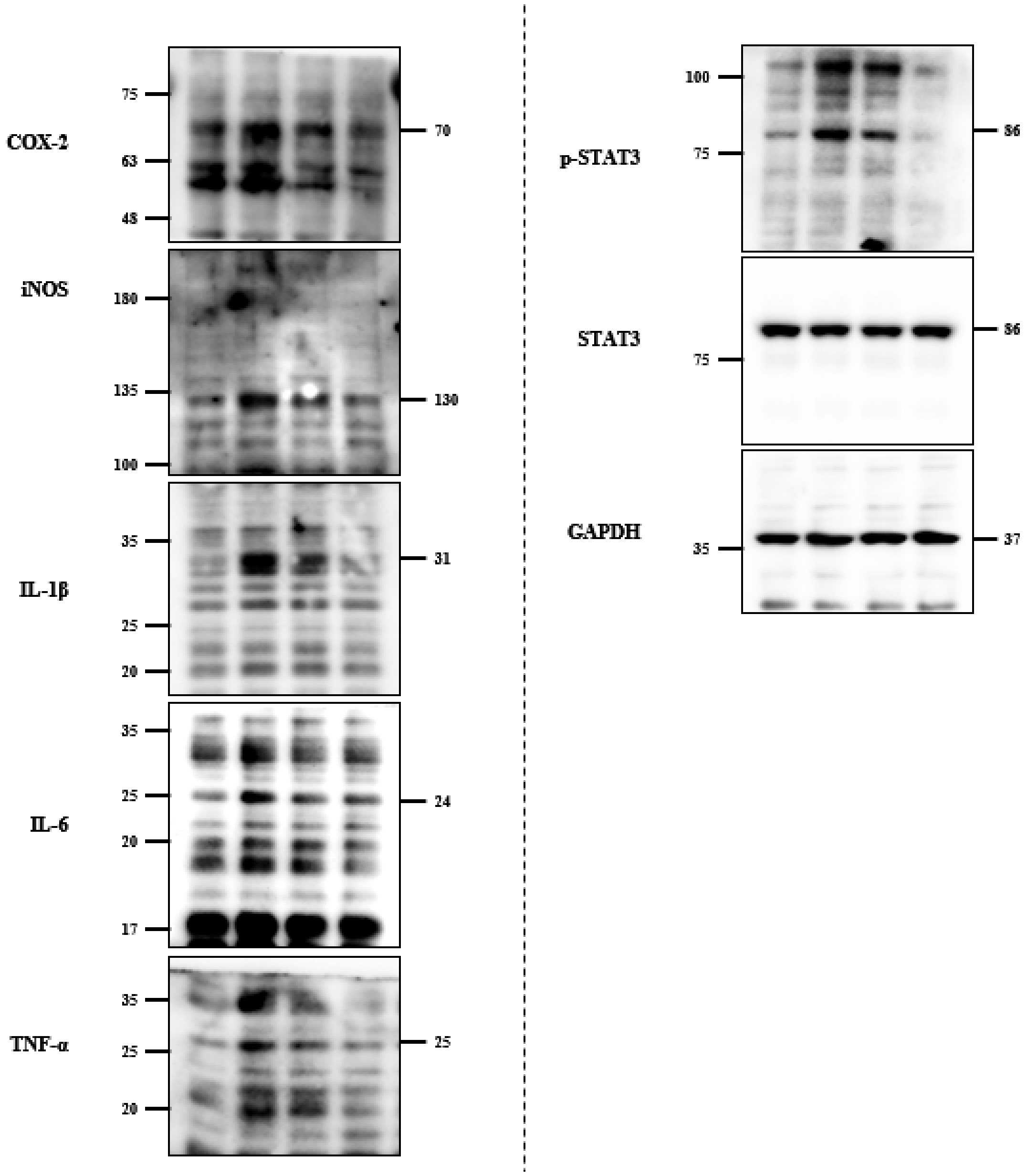
COX-2
75
63
48
70
iNOS
180
135
100
130
IL-1β
35
25
20
31
IL-6
35
25
20
17
24
TNF-α
35
25
20
25
p-STAT3
100
75
86
STAT3
75
86
GAPDH
35
37

Figure 6A

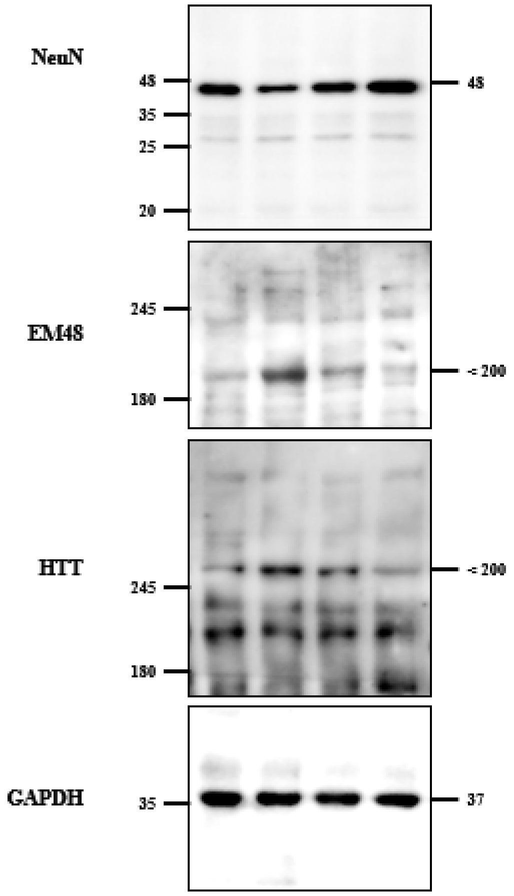

Figure 6G

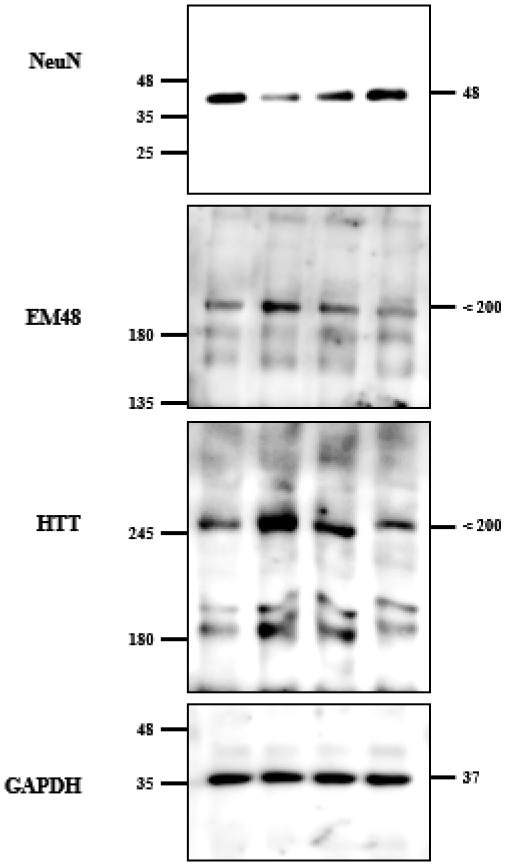

Supplementary Data S1. Original images from Western blot assay

Supplementary Data S2

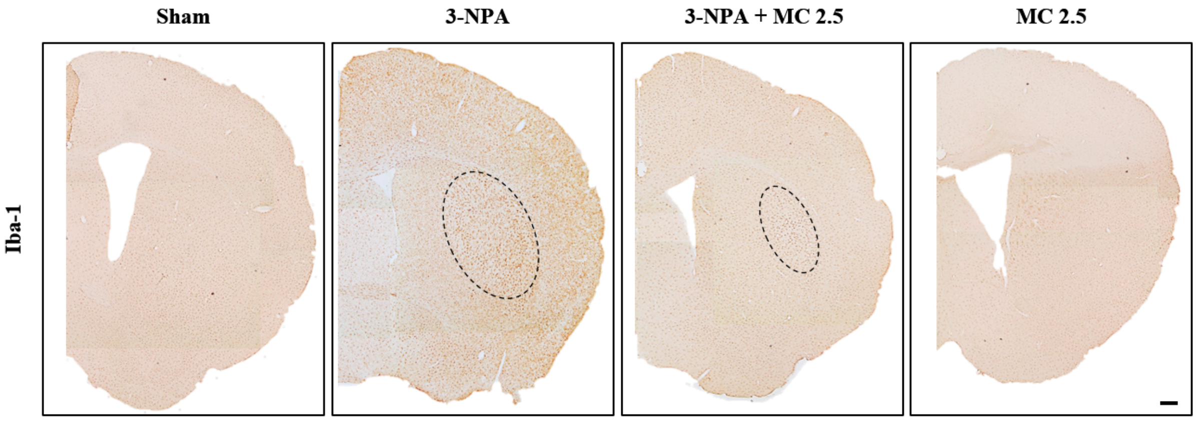

Supplementary data S2. MC inhibits microglial migration and activation in striatum after 3-NPA treatment (a low magnification). Twenty-four hours after the last (5th) 3-NPA treatment, striata from sham, 3-NPA, 3-NPA + MC (2.5 mg/kg/day), and MC (2.5 mg/kg/day) groups were used to investigate the levels of migration of microglia and infiltration of macrophages. MC prevented the migration and activation of Iba-1 immunoreactive cells by immunohistochemistry (A; n = 5 per group).
